# The Effects of Starch Molecular Fine Structure on Thermal and Digestion Properties of Rice Starch

**DOI:** 10.3390/foods11244012

**Published:** 2022-12-11

**Authors:** Cheng Li, Wenwen Yu, Robert G. Gilbert

**Affiliations:** 1 School of Health Science and Engineering, University of Shanghai for Science and Technology, Shanghai 200093, China; 2 Co-Innovation Center for Modern Production Technology of Grain Crops, Yangzhou University, Yangzhou 225009, China; 3 Department of Food Science & Engineering, Jinan University, Huangpu West Avenue 601, Guangzhou 510632, China; 4 Key Laboratory of Plant Functional Genomics of the Ministry of Education/Jiangsu Key Laboratory of Crop Genomics and Molecular Breeding, College of Agriculture, Yangzhou University, Yangzhou 225009, China; 5 Centre for Nutrition and Food Sciences, Queensland Alliance for Agriculture and Food Innovation, The University of Queensland, Brisbane, QLD 4072, Australia

**Keywords:** whole rice, resistant starch, gelatinization, retrogradation

## Abstract

Whole white rice is a major staple food for human consumption, with its starch digestion rate and location in the gastrointestinal tract having a critical role for human health. Starch has a multi-scale structure, which undergoes order-disorder transitions during rice cooking, and this structure is a major determinant of its digestibility. The length distributions of amylose and amylopectin chains are important determinants of rice starch gelatinization properties. Starch chain-length and molecular-size distributions are important determinants of nucleation and crystal growth rates, as well as of intra- and intermolecular interactions during retrogradation. A number of first-order kinetics models have been developed to fit starch digestograms, producing new information on the structural basis for starch digestive characteristics of cooked whole rice. Different starch digestible fractions with distinct digestion patterns have been found for the digestion of rice starch in fully gelatinized and retrograded states, the digestion kinetics of which are largely determined by starch fine molecular structures. Current insights and future directions to better understand digestibility of starch in whole cooked rice are summarized, pointing to ways of developing whole rice into a healthier food by way of having slower starch digestibility.

## 1. Introduction

Whole rice is the main staple food for a significant fraction of the world’s population, especially in Asia and Africa. It supplies many nutrients, including starch, protein and lipids. Starch is the most abundant nutrient in rice, and supplies more than 40% of human daily energy world-wide. The rate and location of starch digestion in the human gastrointestinal tract is critical for human health. If starch is rapidly digested once entering the duodenum, it can cause a large fluctuation in the postprandial glycemic response, which is a risk factor for the development of many chronic diseases such as type 2 diabetes [1]. On the other hand, starch which is slowly digested through the whole gastrointestinal tract results in a stable postprandial glycemic response and supplies sustainable energy [1]. There is frequently a small amount of starch which is resistant to digestion in the human upper gastrointestinal tract, and this starch is termed “resistant starch” (RS). RS can enter the colon and be fermented by gut microbiota into metabolites such as short-chain fatty acids (SCFAs). SCFAs can help prevent or mitigate many intestinal diseases (including diabetes and colo-rectal cancer), promote mineral absorption, control body weight, and reduce blood fat [2,3].

Whole rice grains are almost always consumed by humans following dehulling, polishing and cooking, although a relatively small number of people consume unpolished rice (“brown rice”) in their diets. Development of whole rice or rice-based products with more slowly digestible starch and higher resistant starch content is important for the rice industry, as it would improve public health. However, cooked polished whole rice grains have undergone a high degree of starch gelatinization during cooking, and also there is the lack of a physical barrier to digestive enzymes because of what is removed or degraded by dehulling and polishing.

During the cooking of rice, the starch undergoes order-disorder structural transitions due to the solvation of orthorhombic nanocrystals [4], which affect starch digestibility. Raw rice starch in a whole grain is inherently difficult to digest, due to the presence of the hull and of stable ordered structures such as lamellae, crystalline regions (with an orthorhombic conformation shown using XRD) and granules [5,6,7]. These ordered structures are very stable; this has evolved in Nature for storage starch in a seed, because this starch will only degrade when the plant releases appropriate enzymes for germination, e.g., when the seed is in moist soil. However, there are essentially no human cultures whose diet includes significant amounts of ungelatinized (raw) rice starch. The crystalline structure is largely destroyed during cooking, giving cooked whole white (polished) rice a high glycemic index (GI) and minimal amount of RS [8,9]. After cooking, rice usually is allowed to slowly cool, which changes fully gelatinized starch from an amorphous state to somewhat more ordered structures through both intra- and intermolecular interactions [10,11], thus reducing starch digestibility compared to freshly cooked whole rice [12]. The retrogradation process can also promote the formation of types 3 (retrograded starch) and 5 (amylose-lipid complexes) RS [13,14]. Therefore, controlling starch order and disorder structural transitions during the preparation of cooked whole rice is important for improving the digestibility of its starch.

Starch multi-scale structures, e.g., molecular fine structures (notably the chain-length distributions of both amylose and amylopectin), and, at a higher structural level, microcrystalline domains, are major controllers of starch physicochemical and digestion properties [15]. As discussed in this review, many new insights have been gained over recent years about the relations between rice starch fine molecular structure, gelatinization, retrogradation and digestibility of cooked whole polished rice. One significant advance is that mathematical models have been developed to reduce the whole amylose and amylopectin chain-length distributions (CLDs) to a few biologically meaningful parameters [16,17], which enables a correlation analysis to be carried out between amylose and amylopectin CLD parameters and starch physicochemical and digestion properties: together with the history of starch formation in the organism, these control higher-level properties such as fraction and type of crystallinity, spatial organization of crystalline and amorphous regions, and so on. This analysis has shown that the amounts and distributions of amylose short to intermediate chains (degree of polymerization, DP, ~100–1500) are the major structural features controlling the rice starch retrogradation rate and the digestion rate of long-term retrograded rice starch [12]. Having relatively more and longer amylopectin intermediate chains results in a slower starch digestibility of cooked polished whole rice (at typical rice-to-water cooking ratios) [18]. An amylose content (AC) of ~20% can result in a slow nucleation rate during long-term rice starch retrogradation [19]. Smaller amylopectin molecules and longer amylopectin external chains can promote the development of both inter- and intramolecular interactions during the long-term retrogradation process, possibly due to their high flexibility [20]. Rice amylopectin internal chain segments (i.e., between two branch points) can participate in the formation of intermolecular interactions during retrogradation, and therefore longer amylopectin internal chains can promote the formation of a harder rice-starch hydrogel with a denser microstructure [21]. Generally, the appearance of hexagonal nanocrystals happens during the starch retrogradation process [22].

This review summarizes recent understandings on starch order-disorder structural transitions during conventional whole rice cooking, and the influence of starch fine molecular structures on these processes. Their influence on starch digestibility in cooked whole polished rice is further critically discussed. To the best of the authors’ knowledge, this is the first review to comprehensively discuss starch gelatinization, retrogradation and digestion in an integrated way for whole polished rice at the molecular level. An overview of these new insights, incorporating previous knowledge on starch structure-property relations, may aid the development of cooked whole polished rices with desirable starch digestion properties. This information indicates ways that more nutritionally beneficial cooked whole polished rice having slower starch digestibility could be developed, with potential improvements in public health.

## 2. Overview of Rice Starch Structural Levels and Their Characterization

### 2.1. First Level—Chain-Length Distribution

**Figure 1:**
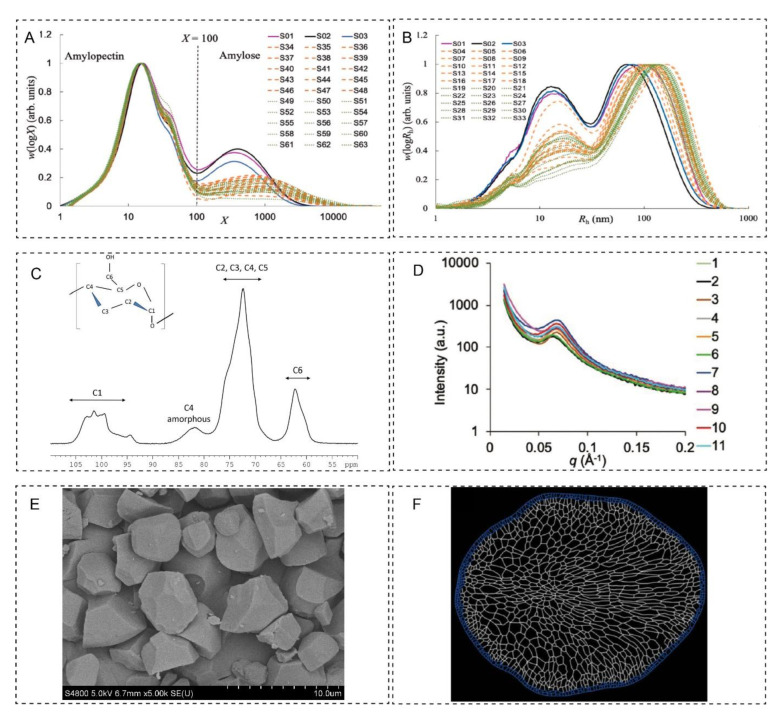
Different levels of rice starch structures. (**A**): level one; CLDs (obtained using SEC on Australian wild and domesticated rices). (**B**): level two; whole molecular size distributions (obtained using SEC on samples of Australian wild and domesticated rice). (**C**): level three; amylopectin double helices (from ^13^C CP/MAS NMR spectrum of japonica rice). (**D**): level four; semi-crystalline growth rings (from small-angle X-ray scattering (SAXS) on wild-type rice varieties from China). (**E**): level five; granules (**E**) (for wild-type rice varieties from China, obtained by scanning electron microscopy). (**F**): level six; cellular structures (from an indica rice (*Oryza sativa* L.) cultivar 9311, from light microscopy). Adapted with permission from Refs. [5, 37,38,39].

Starch has a multi-scale structure, ranging from nanometres to millimetres (Figure 1 and Table 1). Often, six structural levels are defined.

The first level is individual starch chain comprising glucose monomer units connected by (1→4)-α glycosidic linkages, with (1→6)-α branch points. Following enzymatic debranching of the starch sample to quantitatively break every (1→6)-α branch point, the molecular weight or number distribution of this level can be characterized by the following techniques. (a) Size-exclusion chromatography (a widely used technique in polymer characterization) with a mass-sensitive detector, e.g., a differential refractive index (DRI) detector, and a multiple-angle laser light scattering detector for weight-average molecular weight and radius of gyration. With appropriate columns, this technique can be used for any DP range (Figure 1A). (b) Fluorophore-assisted carbohydrate electrophoresis (FACE) with a fluorescence detector, which can be used for DPs below ~150 [26]. (c) High-performance anion-exchange chromatography (HPAEC) with amperometric detection, for DPs below ~60 [27].

Each characterization method has advantages and disadvantages, as follows.

SEC separates by molecular size, not by molecular weight. The column is a solid material with many channels and pores, and smaller molecules diffuse into the smaller pores as well as the larger, and thus elute more slowly than larger molecules. While SEC can characterize any DP range, it requires calibration with standards of known size to convert elution time (or elution volume) into a molecular size, if only a DRI detector is available, which is often the case. With the addition of a light-scattering detector, one has absolute size measurement, this size being the hydrodynamic radius, which is not exactly the same as the geometrical size (the radius of gyration) [28]. It also suffers from “band broadening”: effects such as diffusion in a column during elution mean that a completely monodisperse sample elutes over a finite range after passing through the column, and thus the calibrated size axis is actually an average, termed the SEC-average size. Good SEC columns and instruments minimize, but do not eliminate, this artifact.

FACE works by labelling the individual linear starch polymers (obtained after debranching) with a fluorophore, then separating them by mobility in an electric field; it uses a fluorescence detector. It has very high resolution, with baseline separation between individual DPs, but only works up to DP below ~180 [29]: this covers essentially all amylopectin chains but only a small fraction of amylose chains.

HPAEC (usually with pulsed amperometric detection) [30] can be used with many kinds of carbohydrate oligomers. It can go up to DP~60, but has the disadvantage that there is a mass bias which is complex and laborious to correct in the data analysis to obtain a quantitative distribution [31].

Figure 1A shows typical SEC CLDs: the weight distributions of debranched starches. The (1→4)-α linear chains in starch can be divided into amylose and amylopectin. As is typical, one sees a distinct change at DP ~ 100. For most starches, the amylose chains have DP > 100, while amylopectin almost entirely comprises chains with DP ≤ 100 (Table 1). Amylose content (AC) can thus be measured as the area under the curve of the size distribution of debranched starch with DP > 100 divided by the whole starch CLD area [32,33], which is more accurate than measuring the intensity ratio of the FTIR peaks corresponding to (1→4)-α and (1→6)-α bonds.

Rice can be classified into waxy (0–2%), very low (3–9%), low (10–19%), intermediate (20–25%) and high (>25%) amylose content; these classes can have distinct textural and nutritional properties [34].

Amylopectin CLDs have distinct features, including a global maximum around DP 12 and a shoulder around DP 45, the former being chains confined to a single lamella and the latter being trans-lamellar chains. These amylopectin CLDs can be fitted by a model [17] that notes the existence of several enzyme sets (each comprising one or two of each of a starch synthase, a debranching enzyme and one or two starch branching enzymes), and takes account of the activities of all contributing biosynthesis enzymes to calculate the molecular weight distributions of the chains.

Rice amylose CLDs frequently show only a single broad peak [35], although multiple peaks or shoulders can be observed for some other starches. Amylose CLDs can be parameterized using another model, which is rather different from that for amylopectin while still using activities from multiple enzyme sets, each dominating a DP range [16]. The entire rice starch CLD can thus be parametrized by a small number of biologically relevant parameters (the ratios of various starch biosynthesis enzyme activities) and the mathematical form of the models for amylopectin and amylose biosynthesis. These can be applied to explore rice starch structure-function relations, as exemplified for example in [36].

### 2.2. Second Level—Molecular Size Distribution

The second level structure of rice starch comprises the whole starch molecules, whose molecular size distributions can be characterized by AF^4^ (asymmetirc-flow field-flow fractionation) and by size-exclusion chromatography (Figure 1B). While a number of results for whole-starch size distributions using AF^4^ have been published, it seems that none of those published for typical starch samples have shown that the flow and membrane conditions were such that elution was in so-called Brownian mode [40], i.e., that separation was by molecular size.

At present, the molecular size distributions from SEC among different rice starches can only be compared semi-quantitatively, as these distributions suffer from unavoidable shear scission effects from SEC in the amylopectin region [41]. Two peaks corresponding to the amylose (*R*_h_~20 nm) and amylopectin (*R*_h_~100 nm) molecules are typically observed for ordinary rice starches [37], although mutant rices can show very different behaviour. Different whole-starch structural parameters such as the peak and average *R*_h_ for both amylose and amylopectin molecules can be empirically used for structural comparisons and structure-property relations of different rice starches [42]. The average *R*_h_ value for rice amylose molecules has been reported to range from 13.4 nm to 17.6 nm, and that of rice amylopectin molecules from 66.1 nm to 83.8 nm (Table 1) [12].

### 2.3. Third Level—Nanocrystals

The third level of starch structure comprises the amylopectin orthorhombic nanocrystals, which can be characterized by ^13^C cross-polarized magic angle spinning nuclear magnetic resonance spectroscopy (^13^C CP/MAS NMR) (Figure 1C) and X-ray diffraction (XRD). ^13^C CP/MAS NMR can supply information on the proportion of amorphous, single and double helical material in starch [43]. XRD provides information on the crystal structure of rice starch [44,45].

### 2.4. Fourth Level—Growth Rings

The fourth level of rice starch structure comprises the alternating semi-crystalline and amorphous growth rings, which can be characterized by small angle X-ray scattering (SAXS) (Figure 1D). SAXS can measure the variations in the distribution of electron density between the starch amorphous and crystalline regions, which gives information on the lamellar ordering, fractal nature, lamellar thickness and lamellar thickness polydispersity when fitting SAXS curves with various mathematical models. Typically, SAXS curves for rice starches show a low-*q* power law decay and a lamellar peak around 0.06 Å^−1^ [46]. The SAXS scattering peak is controlled by the periodic arrangement of amylopectin semi-crystalline lamellae, and the overall scattering pattern can be fitted with a combination of a power-law function and a Gaussian-Lorentzian peak function [47]. Different parameters, including the peak area, the full width at half maximum of the peak and the power-law exponent can be obtained from the fitting, giving the ordering of amylopectin semi-crystalline lamella, polydispersity of the lamellar thickness and surface/mass fractal nature of the starch granules, respectively [48]. A one-dimensional linear correlation function can be further applied to obtain information on the thickness of amorphous (*d*_a_) and crystalline (*d*_c_) lamellae [49]. Typically, the overall lamellar distance *d* range from 8.62 to 9.11 nm, *d*_a_ from 3.63 to 3.75 nm, and *d*_c_ from 4.99 to 5.35 nm for rice starches (Table 1) [24]. The dimensions are different from those in the nanocrystals (forming the starch granules), found in normal maize starch [50].

### 2.5. Fifth Level—Granules

The fifth level of rice starch structure comprises the starch granules, which can be characterized quantitatively by a variety of particle-size measuring devices for the appropriate size range, and qualitatively by scanning electron microscopy (SEM). Native rice starch has a polyhedral shape (Figure 1E), which is possibly derived from the tight packing of starch granules in plastids during rice starch biosynthesis [51]. Rice starch shows a unimodal granule size distribution, with a granule size from about 2 to 8 μm (Table 1) [23].

### 2.6. Sixth Level—Cellular Structure

The sixth level of rice starch structure is frequently referred to as the cellular or grain structure (Figure 1F). In the rice endosperm, starch is biosynthesized in storage cells, which have a complex 3D architecture and many non-starch chemical components: protein, lipids and non-starch polysaccharides. Important perspectives of cellular or grain structures include (1) the spatial distribution of cellular components; (2) cell wall architecture and chemical composition; (3) the protein matrix and (4) lipids and polyphenols (Table 1), which can all interact with starch during food processing and during digestion in the human gastrointestinal tract, and significantly change starch physicochemical and nutritional properties [25].

## 3. Starch Order-Disorder Structural Changes during Conventional Cooking of Whole Rice

Levels 1 and 2 starch structures are normally referred to as amorphous structures, while higher levels of native starch structures (level 3 to 6) can be referred as crystalline structures, due to the presence of amylopectin double helices. Conventional cooking of whole dehusked (i.e., white) rice involves the cooking with certain amount of water until the disappearance of the white core in the grain, followed by cooling to various extents (depending on the culture and region) prior to human consumption. During this process, rice starch undergoes gelatinization and then retrogradation, involving starch structural order-disorder transitions, which have been shown to be critical for starch digestibility in cooked whole rice.

### 3.1. Starch Fine Molecular Structure and Gelatinization during Cooking

As stated, during the cooking of whole rice, starch (mainly the amylopectin component) undergoes an order-to-disorder phase transition, which greatly disrupts starch semi-crystalline structures, the extent of which depends on the cooking method. Starch gelatinization involves disruption of the hydrogen bonding in the crystal, which can be followed by events such as starch-granule swelling and amylose leaching, as well as the disruption of amylopectin double helices [52]. (1) At the beginning of starch gelatinization, free water enters the amorphous growth rings, wherein the rice starch granules begin to swell and amylose molecules begin to leach out [53,54]. (2) The disruption of amylopectin crystallites is only initiated when the amorphous background is sufficiently swollen to cause sufficient stress through connecting molecules from amorphous to crystalline regions. (3) At this stage, amylopectin helix-helix side chains are first dissociated, followed by the unwinding of amylopectin double helices [52].

Starch molecular fine structures are important factors in determining rice starch gelatinization properties, including by affecting the types and ordering of nanocrystals (Table 2). According to the amylopectin cluster model, amylopectin A (~DP 6–12) and B1 (~DP 13–24) chains can form double helices in the semi-crystalline lamella of starch granules [55]. Therefore, longer and more thermally stable amylopectin double helices are frequently related to the presence of longer amylopectin A and B1 chains (Table 2) [56,57]. Amylopectin A chains can be further divided into A_cltr_ chains (~DP 9–12, which preferentially form double helices) and A_fp_ chains (~DP 6–8, which are too short to form double helices) [58]. The amount of amylopectin A_cltr_ chains is positively associated with starch gelatinization temperatures (Table 2) [59,60,61]. Recently, it was found that the rice starch gelatinization conclusion temperature was associated with amylopectin trans-lamellar chains (~DP 37–69), possibly due to the formation of long amylopectin double helices among chains protruding from the crystalline lamellae into the adjacent amorphous lamella (Table 2) [62]. Longer inter-block chain length is positively correlated with the gelatinization onset temperature for different starches, possibly due to longer amylopectin internal chain segments being able to have a more ordered alignment of amylopectin double helices in the crystalline lamellae (Table 2) [57,63].

In terms of amylose fine molecular structure, it has been shown recently that AC has an approximately parabolic relationship with rice-starch gelatinization temperatures (Table 2) [64]. That is, either a low or high AC can promote the development of more ordered amylopectin double helices in native rice starch granules. In addition to AC, other features of amylose molecular structures could also play a significant role in determining rice starch gelatinization property, as rice starches with a similar AC often show distinct gelatinization and pasting properties [56]. It has been proposed that longer amylose intermediate chains can interact with amylopectin molecules forming double helices in the crystalline lamella, increasing the heterogeneity of amylopectin double helices, as indicated by the increasing DSC gelatinization temperature range (*T*_c_ − *T*_o_) of rice starches (Table 2) [24,62]. This hypothesis is supported by earlier studies which show that the amount of starch double helices in native starch granules is significantly higher than the starch crystallinity from the long-range order of amylopectin double helices [67]. For amylose biosynthesis, granule-bound starch synthase isoform I (GBSSI) has been hypothesized to be immobilized in amylopectin crystalline lamellae, making use of amylopectin branches or malto-oligosaccharides as primers to synthesize amylose molecules [68]. Therefore, there is also the possibility that amylopectin can interact with amylose through their –OH groups in the crystalline lamellae during starch-granule biosynthesis. It has also been proposed that short amylose chains (DP 100–150) can interact with amylopectin side chains in the amorphous lamellae [69], which might also contribute to starch gelatinization characteristics.

### 3.2. Importance of Starch Fine Molecular Structures for Starch Retrogradation during Cooling of Cooked Dehulled Rice Grains

After whole rice is cooked, retrogradation occurs when it is cooled. Three main steps are involved during this process: (1) nucleation, (2) crystal growth and (3) maturation [70]. The nucleation step is thought to involves the initial interactions or entanglements of starch molecules during retrogradation, which is the rate-limiting step and has a rate constant 10^8^ times slower than that for crystal growth [19]. Crystal growth involves the propagation of hydrogen bonds among starch molecules from the nucleation sites (generally forming hexagonal crystals), and the maturation step is the rearrangement and perfection of retrograded crystallites, due to the high tendency of starch hydroxyl groups to form hydrogen bonds and the existence of strong electrostatic attractions between adjacent starch molecules. A sequence of physical alterations of cooked whole rice occurs with the progression of starch retrogradation, including the appearance of B-type crystallites, exudation of water and increased hardness [71,72].

Rice starch fine molecular structures are the main driving factors determining the nucleation and crystal growth rate during retrogradation, when all higher levels of starch structures are destroyed during gelatinization. Amylose has a rapid retrogradation rate due to its high mobility, which can thus result in the development of the skeleton of a starch hydrogel network, within which amylopectin molecules retrograde with a much slower rate [70,73,74,75,76,77]. Crystallites formed during amylose short-term retrogradation can further provide crystal seeds for the long-term retrogradation of amylopectin [78]. For example, amylose fine molecular structures have been shown to be the dominant factor in terms of determining rice amylopectin long-term retrogradation processes: an AC above 20% can increase the nucleation rate for long-term amylopectin retrogradation (Table 2) [19]. Furthermore, the presence of amylose with a larger molecular size can increase the nucleation rate for short-term rice-starch retrogradation, possibly due to its high mobility (compared to amylopectin molecules) and dense nucleation sites (Table 2) [65]. Relatively more amylose short-to-intermediate chains or longer amylopectin intermediate chains can further increase the rate and extent of crystallization of rice amylopectin molecules [65]. This is supported by a study on corn starch, which showed that shortened amylose molecules subjected to sonication (noting that sonication can cause molecular degradation) became more readily leached out from starch granules during gelatinization; these leached molecules were postulated to align into nuclei and thus to accelerate the retrogradation process [79].

Starch fine molecular structures can also determine interaction patterns (i.e., intra- versus intermolecular interactions) among starch molecules during retrogradation [20]. Different interaction patterns could result in retrograded starch with distinct physical structures, and ultimately determine physicochemical and nutritional properties of cooked whole rice. Amylopectin commonly forms short double helices via intramolecular interactions, due to the high steric hindrance of (1→6)-α glycosidic linkages [20]. However, there is also evidence showing that longer amylopectin external chains and smaller amylopectin molecules can form both inter- and intramolecular interactions during the long-term retrogradation process, due to their high flexibility (Table 2) [20]. Rice amylopectin internal chain segments (i.e., between two branch points) can also participate in the formation of intermolecular interactions during retrogradation, and thus longer amylopectin internal chains can promote the formation of a harder rice-starch hydrogel with a denser microstructure [21]. On the other hand, amylose can form long-range double helices via both intra- and intermolecular interactions [22]. A recent study has further shown that amylose can interact with amylopectin molecules to form short intermolecular double helices during retrogradation [11].

## 4. Digestibility Measurements and Starch Digestibility in Cooked Whole Rice

### 4.1. Digestion Kinetics

In vitro digestion models simulating human gastrointestinal tract are widely applied to study rice starch digestibility [80], as they are cost effective, efficient and do not have ethical issues compared to human or animal studies. There is a wide range of ways of such models, ranging from simple monitoring of glucose concentrations in a “one-pot” set-up though to systems involving complex reactor systems and protocols. When cooked whole rice or other starch-based foods are digested with an excess of starch digestive enzymes, including pancreatic α-amylase and/ or amyloglucosidase, the extent of starch digestion can be plotted against time as a starch digestogram. In many cases, it is possible to reduce the rice starch digestogram into a few kinetics-based parameters, based on a model for the corresponding biological process. This is of significance, in terms of directly presenting the digestion profile and enabling a structure-digestion correlation analysis for a better understanding of starch digestive characteristics. For a homogenous food system, a single first-order kinetics model (SK) is often enough for to fit the whole in-vitro starch digestogram into a single rate constant (Table 3) [81,82,83]. However, the simple SK model cannot reveal mechanistic details of heterogenous starch digestograms with more than one starch digestible fraction (i.e., fast and slow digestion steps). A logarithm of slope plot (LOS), involving numerical derivatives of mathematical transformations of a plot of glucose concentration against time, has been developed to identify the number of starch digestible fractions with distinct rate constants in digestion systems with a sequential digestion pattern (Table 3) [84,85]; it is noted that the resulting parameter values need to be refined by non-linear least squares (NLLS) fitting to avoid the high uncertainty when evaluating numerical derivatives [86].

A sequential digestion pattern is defined as that when the digestion of a slowly digestible starch fraction (SDF) only starts when the digestion of essentially all rapidly digested fractions (RDF) are completed. Seeing more than one straight-line region in an LOS plot indicates more than one starch digestion step. For heterogenous food systems containing simultaneously digestible fractions, e.g., a partially gelatinized starch with both melted (RDF) and intact (SDF) double helices, a parallel first-order kinetics model (PK) should be applied (Table 3) [87]. Another model, a combination of parallel and sequential first-order kinetics (CPS), has been developed to differentiate the digestion pattern (i.e., sequential, parallel or a combination of both) in heterogenous digestion systems [65]. A consecutive reaction kinetics model (CRK) has also been developed recently to distinguish the relative contributions of the enzymatic binding and catalysis steps in the overall starch digestogram (Table 3) [88].

**Table 3:** Kinetics models for understanding starch digestion property.

Kinetics Model	Equation	Parameters	Advantages and Applications	Disadvantages and Limitations	Reference
Single first-order kinetics (SK)	$C\left(t\right)=C_{0}+\left(C_{\infty }-C_{0}\right)\times \left(1-e^{-kt}\right)$	*C*$(t):\;\mathrm{total}\;\mathrm{amount}\;\mathrm{of}\;\mathrm{starch}\;\mathrm{digested}\;\mathrm{at}\;\mathrm{digestion}\;\mathrm{time}\;t;\;C_{0}$$:\;\mathrm{starch}\;\mathrm{digested}\;\mathrm{at}\;t=0;\;C_{\infty }$: starch digested amount at infinite time; *k* is a pseudo-first-order rate constant.	Being able to quantify the whole digestogram into a single rate constant.	Less capable of fitting to multi-phase starch digestograms.	[81]
Logarithm-of-slope plot (LOS)	ln $\frac{dC_{t}}{dt}=\ln[k(C_{\infty }-C_{0})]-kt$	Parameters are the same as those of the SK model.	Being able to identify the number of starch digestible fractions and fit digestograms with a sequential digestion pattern.	Only suitable for fitting to multi-phase starch digestograms with a sequential digestion pattern.	[84]
Parallel first-order kinetics (PK)	$C\left(t\right)=C_{0}+C_{1\infty }\left(1-e^{-k_{1}t}\right)+C_{2\infty }\left(1-e^{-k_{2}t}\right)$	*C*_0_ is the starch digested amount at *t* = 0; *C*_1∞_ and *C*_2∞_ are the maximum starch digested amount at very long time for digestible fractions 1 and 2, respectively; *k*_1_ and *k*_2_ are rate constants for the two digestible fractions, respectively.	Being able to fit digestograms with a parallel digestion pattern.	Only suitable for fitting to multi-phase starch digestograms with a parallel digestion pattern.	[87]
Combination of parallel and sequential kinetics (CPS)	$C\left(t\right)=C_{0}+C_{1\infty }\left(1-e^{-k_{1}t}\right)+\mathrm{If}\left(t\geq t_{2\mathrm{start}},\left(C_{2\infty }\left(1-e^{-k_{2}\left(t-t_{2\mathrm{start}}\right)}\right)\right),0\right)+\ldots$	The parameters *C*_0_, *C*_1∞_, *C*_2∞_, *k*_1_ and *k*_2_ are similar to that of PK model. *t*_2start_ is the time when the digestion of slowly digestible fraction commences.	Being able to identify the digestion pattern of different starch digestible fractions.	Less capable of identifying the number of starch digestible fractions	[65]
Consecutive reactions kinetics (CRK)	For the starch substrate: *C*_SS_(*t*) = exp(−*k*_b_*t*) *C*_DP_(∞).	$C_{\mathrm{SS}}\left(t\right)$$,\;C_{\mathrm{ES}}\left(t\right)$$\mathrm{and}\;C_{\mathrm{DP}}\left(t\right)$$\mathrm{are}\;\mathrm{the}\;\mathrm{ratios}\;\mathrm{of}\;\mathrm{starch}\;\mathrm{amount}\;\mathrm{in}\;\mathrm{substrate},\;\mathrm{enzyme}-\mathrm{starch}\;\mathrm{complex}\;\mathrm{and}\;\mathrm{digested}\;\mathrm{starch}\;\mathrm{product}\;\mathrm{at}\;\mathrm{time}\;t;\;C_{\mathrm{DP}}(\infty )$ is the starch digested ratio after infinite digestion time; *k*_b_ and *k*_cat_ are the rate constants for the formation of enzyme-substrate complex and of digestion product, respectively.	Being able to differentiate the enzymatic binding and catalysis step in starch digestograms.	n.a.	[88]
	For the enzyme-starch complex: $C_{\mathrm{ES}}\left(t\right)=\frac{k_{b}\;C_{\mathrm{DP}}(\infty )}{k_{\mathrm{cat}}-k_{b}}\left(e^{-k_{b}t}-e^{-k_{\mathrm{cat}}t}\right)$				
	For the digested starch product: $\;C_{\mathrm{DP}}\left(t\right)=C_{\mathrm{DP}}(\infty )\times \left(1-\frac{k_{\mathrm{cat}}\;e^{-k_{b}t}-k_{b}e^{-k_{\mathrm{cat}}t}}{k_{\mathrm{cat}}-k_{b}}\right)$				

Note: Parameters obtained from the digestion kinetics models can be applied for the correlation analysis with starch structural parameters in order to understand the structural basis for the starch digestive characteristics in cooked whole rice (as discussed in the main text). n.a. is not available.

### 4.2. The Importance of Starch Molecular Fine Structure for Starch Digestibility of Cooked Whole Rice

The major patterns in the starch digestibility of cooked whole rice depend on whether the starch is in a raw, gelatinized or retrograded state. Raw starch is difficult to digest compared to gelatinized starch, due to the presence of crystalline and granular structures, as summarized in Section 2. Therefore, limiting the degree of starch gelatinization could be potentially investigated as a strategy to reduce the starch digestibility in cooked whole rice, similar to that done with pure isolated waxy rice starch [89] and bread [90]; however, it is essential also to examine the effects of this on palatability (people will not eat healthy food if it has unacceptable mouth-feel). Digestibility studies of raw rice starch are really only applicable to its availability in animal feed, since humans rarely eat a significant amount of raw rice, because it is unpalatable. It is also noted that use of raw rice as animal feed is rare, because of its expense, and only occurs when the grains are badly damaged. Therefore, the digestibility kinetics of raw rice starch is not further discussed in the current review.

When completely gelatinized, all starch crystalline structures are melted, which can substantially increase the starch digestibility of cooked whole rice. Before the consumption of cooked rice, either short-term (e.g., a few minutes to half an hour cooling at room temperature) or long-term (storage in a refrigerator overnight or for few days), retrogradation is common. Differences in starch digestibility after gelatinization are thus determined by factors relating to starch fine molecular structures and their tendency to retrograde: a higher extent of retrogradation will result in a slower starch digestion rate [12,91,92].

The transition from disordered to ordered starch structures during retrogradation has a critical role in determining starch digestibility in cooked whole rice. There are two simultaneous starch digestion fractions for fully gelatinized rice starches [66], with the rate constants of the slowly digestible fraction significantly correlated with rice starch molecular sizes; larger amylopectin molecules tend to have shorter branches, which supply more non-reducing ends for amyloglucosidase, resulting in a higher starch digestion rate. The number of starch digestible fractions is determined by the amount of amylose short to intermediate chains as well as the overall molecular size of rice starches after short-term retrogradation: larger starch molecules and fewer amylose short to intermediate chains can result in two starch digestible fractions with a combination of parallel and sequential digestion patterns [65]. Only a single first-order kinetics phase was observed for rice starches after long-term retrogradation, and rice starches with more amylose short to intermediate chains have a faster retrogradation rate and are prone to form a hydrogel with a denser matrix, which can act as a physical barrier for the diffusion of starch digestive enzymes [12,65]. Retrograded starch double helices could also inhibit the catalytic efficiency of α-amylase through a mixed competitive and non-competitive pattern [93].

## 5. Future Directions

There is moderately good understanding of the effects of starch fine molecular structures on starch gelatinization, retrogradation and digestion properties in whole cooked rice, but many questions remain to be further investigated. For example, cooking conditions (including the time and storage conditions between cooking and consumption) are also important for starch digestibility in cooked whole rice [94]. It is unclear if, and how, starch multi-scale structures and cooking conditions together affect starch digestibility in whole rice. A recent study has investigated the combined effects of starch fine molecular structures and rice-to-water cooking ratios on starch digestibility in cooked whole rice, and found that relatively more and longer amylopectin intermediate chains can cause a slower starch digestion rate, but only with rice-to-water ratios between 1:1 and 1:1.2 [18]. This suggests that both starch structure and cooking conditions play a significant role in determining starch digestibility in whole rice. Conditions such as cooking temperature and time should be considered in the future, together with starch fine molecular structures, in terms of determining rice starch digestibility. In addition, starch is present with protein, lipids and other non-starch polysaccharides (level 6 structure) within the whole rice grain. Although the importance of some of these components (e.g., endogenous proteins [95]) in determining rice starch digestibility has received some attention, there is still much more to be investigated with respect to the importance of these components, as well as the 3D cell architecture, in determining starch digestibility in whole rice. For instance, would and how do starch, protein, lipids and non-starch polysaccharides interact during whole rice cooking? Would they form binary, ternary or even quaternary complexes? How do these complexes affect the starch digestibility?

Freshly cooked white rice is digested very rapidly unless protected by other food components. For example, adding red-grape polyphenols during cooking can increase the RS content in cooked whole white rice [96]. More food additives could be explored in the future to reduce the starch digestibility in cooked white rice. All these questions remain to be further investigated to better understand starch digestion in cooked whole rice.

## 6. Conclusions

Many new insights have been obtained on the importance of starch fine molecular structures in determining the starch gelatinization, retrogradation and digestibility of cooked whole rice. Starch has a complex multi-scale structure in whole rice grains, which undergoes order-disorder structural transition during rice cooking. Amylose chain-length distributions, as well as the length of amylopectin chains, are significant factors in determining rice starch gelatinization properties. Amylopectin and amylose with different CLDs and molecular size distributions are involved in determining the nucleation and crystal growth rates, as well as inter- and intramolecular interactions during retrogradation.

A number of first-order kinetics models have been developed for fitting starch digestograms, which offer a powerful tool to understand the structural basis for starch digestion properties in cooked whole rice. Different numbers of starch digestible fractions with different digestion patterns have been found for the digestion of rice starch in the fully gelatinized and retrograded states, which are partly determined by starch fine molecular structure. The combined effects of starch fine molecular structures, other ingredients in whole rice (such as lipids, which could form amylose-lipids in cooked whole rice as a type of resistant starch) and cooking conditions on the starch digestibility of cooked whole rice should be a focus of research in the future. Considering the popularity of cooked whole rice as a staple food, the information summarized in the current review can help the food industry to develop whole rice into healthier food products with slower starch digestibility, as long as there is no significant diminution of palatability.

## Figures and Tables

**Table 1:** Different levels of starch structure.

Level	Type of Structure	Main Parameters	Range	Reference
1st	Individual chains	Amylopectin CLD	DP ≤ 100	[12]
		Amylose CLD	DP > 100	
2nd	Whole starch molecules	Amylopectin *R*_h_	Average *R*_h_ 66.1–83.8 nm	[12]
		Amylose *R*_h_	Average *R*_h_ 13.4–17.6 nm	
3rd	Double helices	FTIR 1045 cm^−1^/1022 cm^−1^	0.59 to 0.67	[23]
4th	Growth rings	*d*	8.62–9.11 nm	[24]
		*d*_a_	3.63–3.75 nm	
		*d*_c_	4.99–5.35 nm	
5th	Granules	Granule size	2–8 μm	[23]
6th	Cells and grains	Spatial distribution of cellular components.		[25]
		Cell wall architecture and chemical compositions;	n.a.	
		Protein matrix;		
		Lipids and polyphenols		

Note: n.a.: not available.

**Table 2:** Relations between rice starch fine molecular structures with its gelatinization, retrogradation and digestion properties, with possible mechanisms proposed.

Property	Relations to Starch Fine Molecular Structures	Proposed Mechanisms	References
Gelatinization	Longer amylopectin A and B1 chains result in higher gelatinization temperatures.	Longer amylopectin A and B1 chains form longer and more stable double helices.	[56,57]
	The amount of amylopectin A_cltr_ chains is positively associated with starch gelatinization temperature.	Amylopectin A_cltr_ chains preferentially form double helices.	[61]
	Gelatinization conclusion temperature is associated with the amount of amylopectin trans-lamellar chains (DP ~37–69).	Amylopectin trans-lamellar chains can form long amylopectin double helices.	[62]
	Amylopectin inter-block chain length is positively correlated with starch gelatinization onset temperature.	Longer amylopectin internal chain segments allow a more ordered alignment of amylopectin double helices.	[63]
	AC has an approximately parabolic relationship with rice starch gelatinization temperatures.	Either a low or high AC can promote the formation of more ordered amylopectin double helices.	[64]
	Longer amylose intermediate chains can increase the gelatinization temperature range of rice starches.	Longer amylose intermediate chains can interact with amylopectin molecules into double helices.	[24,62]
Retrogradation	AC > 20 % can increase the nucleation rate for long-term amylopectin retrogradation.	Crystallites formed by amylose provide crystal seeds for amylopectin long-term retrogradation.	[19]
	Amylose with a larger molecular size promotes the nucleation rate for short-term rice starch retrogradation.	Larger amylose molecules have a higher mobility and denser nucleation sites.	[65]
	Longer amylopectin external chains and smaller amylopectin molecules can have both inter- and intramolecular interactions.	Longer amylopectin external chains and smaller amylopectin molecules have higher flexibility.	[20]
Digestion	Larger rice amylopectin molecules have a faster digestion rate at the fully gelatinized state.	Larger amylopectin molecules have shorter branches, supplying more non-reducing ends for amyloglucosidase.	[66]
	Rice starches with more short to intermediate amylose chains have a slower starch digestion rate in the retrograded state.	Amylose short to intermediate chains are prone to retrograde.	[12]

## Data Availability

Data is contained within the article.
